# Biliary microbiota and metabolic reprogramming in gallstone-associated gallbladder cancer: evidence from bile, stones, mucosa and tumor tissue

**DOI:** 10.3389/fonc.2026.1925531

**Published:** 2026-09-25

**Authors:** Dongzhe Li, Hongpeng Guo, Junjie Zhang

**Affiliations:** 1 Department of General Surgery, Central Hospital Affiliated to Shenyang Medical College, Shenyang, Liaoning, China; 2 Key Laboratory of Microecology-Immune Regulatory Network and Related Diseases School of Basic Medicine, Jiamusi University, Jiamusi, Heilongjiang, China; 3 Department of Pathology, Central Hospital Affiliated to Shenyang Medical College, Shenyang, Liaoning, China

**Keywords:** bile acids, biliary microbiota, cholelithiasis, chronic cholecystitis, early detection, gallbladder cancer, metabolic reprogramming, multi-omics

## Abstract

Gallbladder cancer (GBC) commonly arises in gallbladders affected by cholelithiasis and chronic cholecystitis, although malignancy develops in only a small minority of patients with stones. This disparity has directed attention to the local conditions created by concentrated bile, stone surfaces, persistent microbes and altered bile acid and lipid metabolism. Evidence in this field is difficult to compare because bile, stones, mucosa and tumor tissue measure different biological processes, while human associations, animal experiments and cross-sectional omics studies are often discussed together. We evaluated microbiological, metabolic and tissue-level studies according to the material examined, disease endpoint and study design, covering gallstone formation, chronic epithelial injury, preneoplastic lesions and established GBC. Salmonella is the most extensively studied microbial candidate, but epidemiological estimates vary with carrier prevalence, detection method and the use of gallstone controls. Fungal findings in GBC currently derive from one exploratory peptide-classification study, whereas separate fungal experiments concern gallstone formation. TMAO has reproducible effects on biliary cholesterol handling and lithogenesis; its relation to GBC is based on cross-sectional plasma measurements and preneoplastic histology in lithogenic-diet mice, without invasive cancer in that model. Single-cell and spatial studies identify epithelial stress, myeloid recruitment, T-cell exhaustion and stromal remodeling in sampled tissues, but precursor lesions remain sparse and pseudotime does not represent clinical follow-up. Bile lipidomics, microbial peptides and circulating markers may aid preoperative assessment when tested against inflammatory gallbladder disease. Distinguishing the material examined and the measured endpoint separates evidence for stone formation, persistent mucosal injury and established cancer, and identifies where paired bile, stone, mucosal and tumor samples can answer specific biological questions.

## Introduction

Gallbladder cancer (GBC) is the most common malignancy of the biliary tract and one of its most lethal. Curative treatment depends on early resection, yet many tumors are detected only after local invasion or incidentally after cholecystectomy. Incidence varies sharply by geography, sex and ancestry (1, 2). Gallstones and chronic cholecystitis are the dominant clinical background, alongside metabolic, reproductive, infectious and environmental exposures (3, 4). Yet only a small proportion of affected patients develop GBC. The unresolved biological question is which local exposures and tissue responses distinguish common gallstone disease from gallbladders that acquire dysplasia or carcinoma.

The gallbladder concentrates hepatic bile and repeatedly exposes its epithelium to cholesterol crystals, bile acids, phospholipids, bilirubin derivatives and mucin. Stone surfaces can retain microorganisms and their extracellular matrix, while obesity, insulin resistance and altered enterohepatic circulation influence bile composition and gallbladder motility (5, 6). Sequencing and multi-omics studies have detected low-biomass microbial signals in bile, stones and mucosa (7, 8). These measurements are vulnerable to contamination, sampling route, antibiotics, drainage and disease-related obstruction (9, 10). The material collected determines the question that can be answered: bile reflects soluble exposures, stones retain adherent communities, mucosa records epithelial contact and injury, and tumor tissue describes established malignant biology.

Early studies largely cataloged taxa or metabolites, whereas newer work combines molecular profiling with functional experiments. Bile lipid and microbial peptide signatures distinguish GBC from gallstone-associated inflammation in selected cohorts (11, 12). Fungal peptide data add a possible diagnostic signal, although the GBC evidence comes from one small exploratory study (13). TMAO and diabetes-related gallbladder studies link intestinal metabolism with lithogenic bile and inflammatory injury. The TMAO mouse model showed hyperplasia and metaplasia and did not produce invasive cancer (14, 15). Salmonella work provides a distinct infection model, but the human epidemiology concerns S. Typhi while the recent perturbation study used S. Typhimurium (16).

Recent reviews have considered gut microbiota across biliary tract cancers, proposed microbial mechanisms in GBC, or combined biliary microbiome, metabolomics and bile-acid biology (17–20). Their broad scope leaves a narrower interpretive problem unresolved: signals measured in feces, bile, stones, mucosa and tumor tissue do not address the same biological event. We therefore compare findings by material examined, disease endpoint and study design. The discussion asks whether the evidence concerns gallstone formation, persistent epithelial injury, established cancer or preoperative classification. Human associations are separated from animal, cell and organoid perturbation experiments (**Figure 1**).

**Figure 1:**
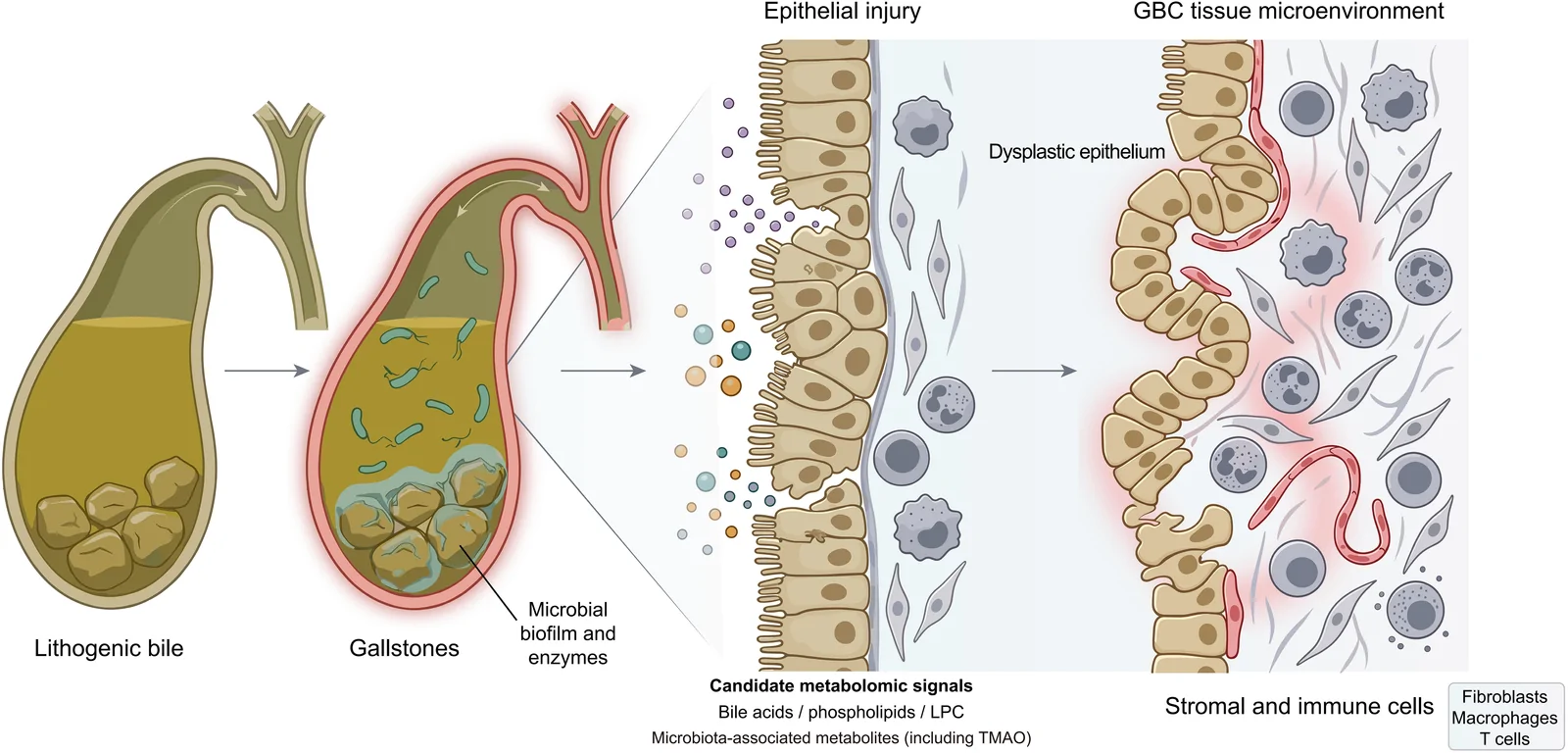
Microbial and metabolic findings across gallstone-associated gallbladder disease. The schematic places lithogenic bile and gallstones alongside microbial biofilms and enzymes, epithelial injury and dysplasia. Candidate metabolomic signals include bile acids, phospholipids, lysophosphatidylcholine (LPC) and microbiota-associated metabolites such as TMAO. TMAO is placed at the stage tested in lithogenic-diet mice, which developed gallstones and preneoplastic histology but not invasive GBC. The final panel summarizes epithelial, immune, stromal and endothelial states in GBC tissue. Arrows organize the reported findings without indicating longitudinal evidence; metabolite labels do not identify microbial producers.

## Literature identification and evidence appraisal

This narrative review was supported by a structured literature search. PubMed was searched using 11 topic-specific strategies. The strategies covered Salmonella and GBC, Salmonella carriage and biofilms, biliary fungi, TMAO, single-cell and spatial studies, biliary microbiome and metabolomics, bile acids and lipids, cholecystitis and dysplasia, recent reviews and low-biomass methods. Crossref was searched for five supplementary topics, and reference lists of systematic reviews and primary studies used in the main comparisons were checked. The PubMed searches yielded 2,530 unique records before topic-level screening. Exact search strings and retrieval counts are provided in **Supplementary Table S1**.

Eligible reports were peer-reviewed human, animal, organoid or cell studies that examined gallstones, chronic cholecystitis, gallbladder preneoplasia or GBC in relation to microbial persistence, biliary or intestinal microbiota, fungal findings, bile acids, lipids, TMAO, tissue response or preoperative detection. Systematic reviews and meta-analyses were used for synthesis and reference checking. Narrative reviews were used for background. Case reports were excluded from general claims, as were studies of other hepatobiliary cancers when GBC results could not be separated and microbiome studies without a gallbladder-related endpoint.

Studies used for the main comparisons were appraised by design, comparator, sample size, replication, direct perturbation and measured endpoint. Evidence was classified as human epidemiology, human molecular profiling, human tissue profiling, animal perturbation, cell or organoid perturbation, systematic review or narrative review. Interpretive weight was recorded as limited, moderate or higher for the stated endpoint. Limited evidence comprised exploratory or small cross-sectional studies without independent replication. Moderate evidence comprised human studies with relevant comparators but residual confounding, or direct perturbation experiments that did not model the corresponding human disease endpoint. Higher weight required replication in relevant human material and endpoint-specific experimental validation. This appraisal is not a GRADE assessment of clinical recommendations.

## From gallstones to chronic cholecystitis and malignant transformation

Cholelithiasis is the most recognizable clinical background for GBC, yet it is an inefficient predictor of cancer at the individual level. It is consistently listed among the best-established environmental and clinical risk factors, whereas conversion from gallstones to GBC remains below 1% (3). This contrast has practical importance. Gallstones identify a gallbladder exposed to lithogenic bile, impaired emptying and repeated mucosal injury. Cholesterol stones arise through biliary supersaturation, impaired motility, mucin accumulation, wall inflammation and systemic metabolic factors; cholesterol excess is only one part of the process (21, 22). Additional information is needed to explain which patients progress toward dysplasia or carcinoma. The relevant biology lies in the tissue response to stones, the microbial communities that stones can support and the metabolic products retained in concentrated bile.

Gallstones may also record a long period of silent exposure before symptoms or cancer are detected. Radiocarbon dating, Fourier-transform infrared spectroscopy and 16S rRNA sequencing of three cholesterol gallstones linked stone age, mineral composition and microbial signal with adjacent normal mucosa, metaplasia or dysplasia (23). The largest stone, associated with dysplastic epithelium, was estimated to have formed over 6 years. Stones associated with metaplasia and normal pathology were estimated to have taken 13 and 12 years to develop, and the reported dormant intervals ranged from 6 to 18 years (23). The stone-level data also showed Corynebacterium enrichment in stones associated with metaplasia or dysplasia and Klebsiella and Escherichia coli in calcium oxalate-rich stones linked to dysplasia (23). Because only three stones were examined, the estimated formation and dormancy intervals are exploratory and cannot define typical gallstone growth rates.

Gallstone disease can leave durable marks on the gallbladder epithelium. In a series of 350 cholecystectomy specimens from patients with gallstones, hyperplasia, metaplasia, dysplasia and carcinoma *in situ* were found in 32.0%, 47.8%, 15.7% and 0.6%, respectively (24). The frequency of metaplasia and dysplasia in this surgical series places these lesions within the pathology of gallstone disease, but the study did not follow individual lesions over time. Clinicopathologic analysis of 447 non-neoplastic gallbladder polyps adds a separate view of injury-related changes. Fibromyoglandular polyps were almost always associated with gallstones and chronic cholecystitis, whereas dysplasia involved only a minority of lesions (25). Gallstone irritation, chronic inflammation, mucin changes, metabolic risk and molecular alterations have all been described in studies centered on gallbladder dysplasia (26). Gallstone-associated injury therefore has variable epithelial outcomes.

Gallstones provide a durable surface for Salmonella persistence. Gallstones are common among chronic S. Typhi carriers, and bile induces extracellular matrix production that supports biofilm growth on cholesterol-rich gallstone surfaces (27). Carriage occurs in a minority of infections, but stone-associated biofilms increase persistence, shedding and antibiotic tolerance (28). This biology explains chronic exposure of the gallbladder epithelium. It does not quantify the subsequent risk of GBC.

Bacteria may also participate in stone formation itself. Bacterial contributions to stones include altered bile composition, changes in the gallbladder environment and production of beta-glucuronidase, mucus and other factors that promote stone formation (29). Biofilm-forming organisms, including Escherichia coli, Klebsiella pneumoniae, Enterococcus spp. and Pseudomonas aeruginosa, have been detected in bile, gallbladder tissue or stones (29). This adds a second microbial route: bacteria can persist on existing stones and may also help create or modify the stone surface that later supports persistence.

Human studies have reported associations between chronic S. Typhi carriage and GBC, but the magnitude changes with the control group. In Chile, 39 GBC cases were compared with 40 gallstone controls and 39 population controls. The adjusted odds ratio for high anti-Vi titers was 4.0 against the combined controls (95% CI, 0.9-18.3). Against gallstone controls alone, the adjusted odds ratio was 3.1 (95% CI, 0.6-17.7). Adjustment was limited to sex and education, and S. Typhi was not recovered from the tested specimens (30). The meta-analysis within that report yielded summary relative risks of 4.6 for anti-Vi seropositivity and 5.0 for bile or stool culture (30). A separate 17-study meta-analysis reported an overall odds ratio of 4.28, with a lower estimate against gallstone controls (OR, 2.71) than against controls without stones (OR, 5.86) and substantial heterogeneity (31). In Shanghai, only six of 2,293 participants were seropositive and no association was detected (32). Dutch record linkage identified nine biliary tract cancers among 16,252 patients with prior salmonellosis (SIR, 1.53; 95% CI, 0.70-2.91) (33). Carrier prevalence, detection method and control selection materially affect the estimate.

Experimental evidence addresses a different question from carriage epidemiology. S. Typhimurium was isolated from GBC tissue, and Salmonella abundance was higher in GBC-associated microbiome data (16). In lithogenic-diet mice, infection produced persistent inflammation, hyperplasia and metaplasia. KDM6B activation was observed in mouse and human GBC tissue, while pharmacological KDM6B inhibition reduced xenograft growth (16). The study links infection with epithelial and epigenetic changes in a stone-forming setting. It does not reproduce human S. Typhi carriage, and the mouse gallbladders did not develop spontaneous invasive carcinoma.

Helicobacter-associated data are less directly linked to GBC, but they are important for the inflammatory stage of gallstone disease. Pooled Helicobacter data from 20 studies linked gallbladder infection with chronic cholecystitis or cholelithiasis, with odds ratios of 3.15 for Helicobacter species and 3.05 for H. pylori (34). Barrier injury has been examined using single-cell and bulk transcriptomics, gallbladder mucosal microbiota profiling, epithelial-cell experiments and a gallstone mouse model. In these experiments, H. pylori exposure increased epithelial permeability and reduced ZO-1 and OCLN expression; CagA was detected in epithelial cells (35). The tested endpoint was barrier injury during gallstone-related inflammation. GBC initiation was not examined.

Staged human tissue transcriptomics place inflammation and metabolism early in the transition from gallstones to cancer. RNA-seq comparison of three normal gallbladders, four gallbladders with gallstone-induced chronic inflammation, five early GBC samples and five advanced GBC samples separated several disease-stage signals (36). The shift from normal gallbladder to chronic inflammation involved inflammatory pathways, lipid metabolism and sex-hormone metabolism. The shift from chronic inflammation to early GBC involved immune activities and cell-connection programs. Advanced disease showed stronger changes in transmembrane transport and cell migration (36). The cohort is small, but the staged design is valuable because it separates inflammation-associated changes from alterations seen after invasive cancer has developed.

Metabolic profiles vary across chronic inflammation, stone disease and GBC. Serum 1H NMR profiling of chronic cholecystitis showed lower glutamine, LDL, VLDL, alanine, branched-chain amino acids, histidine and tyrosine, with higher formate, lactate and 1,2-propanediol (37). Plasma TMAO differed among healthy, gallstone and GBC groups, and lithogenic-diet mice receiving TMAO developed more stones, inflammation, hyperplasia and metaplasia (15). The human groups differed markedly in age; targeted TMAO did not differ significantly between GBC and gallstone disease, and the mouse experiment included no TMAO-only group or invasive carcinoma. Established GBC RNA-seq identified altered LXR/RXR and FXR/RXR signaling (38). Metals and stone composition have also differed between small GBC and cholelithiasis series (39). These results measure chronic inflammation, lithogenesis and established cancer in separate samples and models. They do not support a continuous path from TMAO exposure to GBC.

Bile microbial and metabolic profiles differ between gallstone-associated chronic cholecystitis and cancer. A 16S rRNA gene V3-V4 amplicon sequencing study of 30 patients with GBC and 30 patients with gallstone-associated chronic cholecystitis found disease-associated differences in Streptococcus, Enterococcus, Halomonas, Escherichia and Caulobacter (12). Bile multi-omics studies have also shown that lipid species and microbial peptide signatures can distinguish gallbladder carcinoma from gallstone disease in training and validation cohorts (11). Their value is complementary to tissue pathology and experimental testing: bile contains disease-associated information from both microbes and host metabolism.

For preoperative work, the strongest comparator is an inflamed gallbladder with stones, wall thickening or xanthogranulomatous change. Healthy bile alone cannot test the real diagnostic problem. Studies that compare GBC with gallstone-associated chronic cholecystitis carry more clinical weight than cancer-control contrasts alone (11, 12). Their signals should be reported with imaging findings, operative route, antibiotic exposure, drainage status and stone features, since each factor can change bile composition and microbial yield.

## Biliary microbiota, biofilm and microbial persistence in gallbladder disease

The biliary tract is exposed to intestinal microorganisms but is protected by bile flow, bile salts, mucus, epithelial innate immunity and secretory IgA. Microbial detection in bile is therefore biologically relevant and methodologically sensitive. Low microbial biomass increases the influence of sampling route, perioperative antibiotics, sphincterotomy, drainage and background contamination (8, 9). Across gallstones, non-calculous biliary inflammatory disease and biliary tract cancer, Faecalibacterium loss recurs, whereas Veillonella, Lactobacillus, Streptococcus and Enterococcus increase in several biliary disorders (40). Most datasets examine one disease and one sample type. Bile fluid, gallstones, mucosa and tumor tissue describe different biological processes. **Table 1** appraises the studies most relevant to Salmonella, fungal findings, TMAO and cell-resolved tissue analysis by design, endpoint and limitation.

**Table 1:** Evidence appraisal for Salmonella, fungal findings, TMAO and cell-resolved tissue studies.

Topic	Study design and material	Sample size or model	Finding supported	Main limitation	Interpretive weight and references
Salmonella epidemiology	Human case-control study; anti-Vi serology and microbiology in GBC, gallstone and population controls	39 GBC; 40 gallstone controls; 39 population controls	High anti-Vi titers were more frequent in GBC; adjusted OR 3.1 against gallstone controls	Wide CI, limited adjustment and no S. Typhi recovered from tested local specimens	Limited for GBC association (30)
Salmonella epidemiology	Systematic review and meta-analysis of chronic S. Typhi carriage and GBC	17 studies	Overall OR 4.28; OR 2.71 with gallstone controls and 5.86 with controls without stones	High heterogeneity, mixed assays and weak matching in several studies	Moderate (31)
Salmonella epidemiology	Population-based case-control study in a low-carrier setting	627 biliary tract cancers; 1,037 stone cases; 959 controls	Only six participants were seropositive and no association was detected	Very low exposure prevalence and a broader biliary cancer endpoint	Moderate for a null result in a low-carrier setting (32)
Salmonella epidemiology	National record-linkage cohort after laboratory-confirmed salmonellosis	16,252 exposed patients; 9 biliary tract cancers	SIR 1.53 (95% CI, 0.70-2.91)	Few events and endpoint included several biliary sites	Moderate for longitudinal incidence; limited by event count (33)
Salmonella carriage	Cross-sectional qPCR of bile, stones and gallbladder tissue at cholecystectomy	988 patients	S. Typhi or S. Paratyphi DNA in 3.4%; stones most often positive	No cancer-incidence endpoint	Moderate for carriage detection; no GBC endpoint (47)
Salmonella experiment	Human profiling and culture plus lithogenic-diet mice, cells and xenografts	S. Typhimurium across several models	Colonization, inflammatory and premalignant histology, KDM6B activity and xenograft response	Different serovar from human typhoid studies; no spontaneous invasive GBC in mice	Moderate for injury and established-tumor growth; limited for GBC initiation (16)
Gallstone fungi	Paired 16S and ITS sequencing of bile and stones in chronic cholecystitis	Single disease cohort	Bile and stones carried different bacterial and fungal signals	No healthy or GBC comparator	Limited (45)
Intestinal fungi and stones	Human fecal profiling plus lithogenic-diet mouse perturbation	17 healthy; 19 gallstone; mouse groups generally n=8-10	R. mucilaginosa increased stones and hepatic neutrophil responses in the tested model	Fecal study; fluconazole altered bacteria and caused hepatotoxicity; no GBC endpoint	Limited (46)
Fungal GBC classifier	Bile and plasma fungal peptide profiling with machine learning	Initial series 10 gallstone, 16 GBC and 16 donors; total n=82	Five-peptide panel showed high discrimination in the reported dataset	No culture or ITS confirmation, no epithelial experiment and no external validation	Limited (13)
TMAO and stones	Human cohorts plus mouse and hepatocyte perturbation	573 gallstone and 565 controls; independent second cohort and experimental models	TMAO was associated with stones and increased ABCG5/G8 and biliary cholesterol secretion	Lithogenesis endpoint only	Moderate for lithogenesis; no cancer endpoint (59)
TMAO and hypoxia	Altitude-related human groups plus hypoxic lithogenic-diet mice and hepatocytes	Multiple human groups; many experimental assays used n=3	HIF-1alpha/FMO3/TMAO signaling altered cholesterol handling in lithogenic conditions	Small experimental groups and no cancer endpoint	Limited for the hypoxia context; no cancer endpoint (121)
TMAO and GBC	Human discovery, validation and targeted metabolomics plus mouse experiment	145 humans; chow, lithogenic diet and lithogenic diet plus TMAO mice	Cross-sectional disease-group differences and more stones, inflammation, hyperplasia and metaplasia in mice	Age imbalance, targeted GBC-versus-stone P = 0.08, no TMAO-only group and no invasive cancer	Limited for GBC; moderate for lithogenesis in the tested model (15)
Single-cell tissue states	Cross-sectional scRNA-seq of chronic cholecystitis, primary GBC and metastases	24 samples: 6 chronic cholecystitis, 12 primary GBC and 6 metastases	Distinct epithelial, immune and stromal states across sampled tissues	No within-patient observation from inflammation to cancer	Moderate for sampled tissue states; no longitudinal endpoint (66)
Precursor single-cell study	Cross-sectional scRNA-seq with pseudotime	10 samples: 2 adjacent non-tumor, 2 HGIN and 6 GBC	TOP2A-positive epithelial and macrophage states and CD8 exhaustion differed across groups	Only two HGIN samples; pseudotime is computational ordering	Limited (69)
Spatial precursor study	Spatial transcriptomics with organoid and xenograft follow-up	2 patients; 24 and 32 regions of interest	Patient-specific signaling patterns and SEMA4A-related experimental findings	Two selected tumors cannot define a general disease chronology	Limited (83)
Large GBC single-cell study	scRNA-seq, WES, spatial analysis and perturbation experiments	102 patients; 1,117,245 cells	Extensive primary and metastatic tumor diversity and AREG-related biology	Only 2 low-grade and 1 high-grade lesion among 16 non-tumor gallbladders	Higher for established GBC; limited for precursor lesions (68)

Sequencing has nevertheless changed the scale of microbial detection in inflamed bile. Prospective sequencing of moderate or severe acute cholecystitis detected bacterial and/or fungal material in 31 of 36 bile samples using bacterial 16S rRNA, rpoB and fungal ITS2 assays (41). Conventional culture recovered only 40 of 106 microbes detected by sequencing, and culture missed at least one organism in every polymicrobial sample (41). Anaerobes, oral streptococci, enterococci, non-E. coli Enterobacteriaceae and fungi were particularly likely to be missed. These data address acute inflammation, and their value here is methodological: culture-based bile studies capture only part of the microbial burden in inflammatory disease.

Gallstone disease provides a second reason to study biliary microbes: stones supply a surface on which organisms can attach, grow and tolerate bile stress. Across studies, the pooled summary reported higher ACE and Simpson diversity indices, beta-diversity differences from controls, increased Firmicutes and reduced Proteobacteria (42). Individual genus-level reports more often identified Escherichia-Shigella and Streptococcus enrichment, while Helicobacter findings varied (42). The phylum-level and genus-level summaries came from different cohorts and taxonomic levels. Enrichment of Escherichia-Shigella in one study therefore does not imply higher aggregate Proteobacteria abundance across the pooled literature.

Bacteria may influence stone biology through enzymes, matrix production and bile tolerance. Beta-glucuronidase, phospholipase A2, bile salt hydrolase activity, mucin production and biofilm formation can alter bile chemistry and stone surfaces (43). Newer multi-omics work also indicates that stone type matters. In 29 gallstone patients, type IIB restriction site-associated DNA microbiome sequencing and plasma metabolomics detected bacteria, fungi and one archaeon in bile or stones, with cholesterol gallstone disease linked to altered microbial species and enriched bile acid biosynthesis (44). These findings argue against treating all gallstones as microbiologically equivalent.

Fungal findings in gallstone disease come from bile, stones and feces, and each material has a different meaning. Paired 16S and ITS sequencing in chronic cholecystitis detected bacterial and fungal signals in bile and stones (45). Stones showed stronger bacterial-bacterial, fungal-fungal and cross-kingdom correlations than bile (45). The study had no healthy or GBC comparator, so it supports co-detection on stone surfaces without defining a normal bile mycobiota. Fecal fungal profiling in 17 healthy participants and 19 patients with gallstones identified group differences. Rhodotorula mucilaginosa increased gallstone formation and hepatic neutrophil responses in lithogenic-diet mice, but the experiment addressed intestinal fungi and gallstones, not biliary fungi or cancer. Fluconazole altered bacterial composition and produced hepatotoxicity, which limits its use as a fungus-specific intervention (46).

Stone-associated biofilm biology explains persistence more directly than it explains cancer. Bile induces an O-antigen capsule required for Salmonella biofilm formation on cholesterol-rich gallstone surfaces, and biofilms have been visualized on stones from human carriers (27). Gallstones also increase bacterial persistence, fecal shedding and antibiotic tolerance in experimental carriage models (28). The transition from carriage to GBC remains epidemiological; the Chilean study did not recover S. Typhi from the tested local specimens and its estimate against gallstone controls was imprecise (30). A 2024 Pakistani cholecystectomy series detected S. Typhi or S. Paratyphi DNA in 3.4% of 988 patients, most often in stones, but did not evaluate subsequent cancer (47). Human carriage studies and biofilm models support long-term persistence on stones; GBC risk estimates remain heterogeneous.

Perturbation studies also need to be separated by serovar and endpoint. S. Typhimurium from GBC tissue colonized experimental systems and altered tumor-associated gene expression (16). In lithogenic-diet mice, infection caused inflammatory and premalignant histology; KDM6B inhibition reduced growth of established xenografts (16). These are two distinct endpoints. The gallbladder experiment addresses infection-associated epithelial injury, while the xenograft experiment tests growth of an established tumor. Neither experiment models chronic human S. Typhi carriage or spontaneous invasive GBC.

Helicobacter evidence is more uneven and should be separated by disease stage and species. Pooled data from 20 studies linked gallbladder Helicobacter infection with chronic cholecystitis or cholelithiasis, with odds ratios of 3.15 for Helicobacter species and 3.05 for H. pylori (34). Mechanistic work has added a plausible epithelial route. In gallstone-related models and human mucosal samples, H. pylori CagA was linked to increased epithelial permeability, neutrophil activation and reduced tight junction proteins ZO-1 and OCLN (35). GBC-specific Helicobacter data are less consistent. A case-control study and meta-analysis focused on H. bilis did not find a higher risk in its own GBC cohort compared with cholelithiasis controls, although pooled Helicobacter analyses across studies suggested positive associations (48). A cautious reading is that Helicobacter may contribute to gallstone-associated inflammation in some settings, while its role in GBC remains species- and method-dependent.

Human bile and mucosal studies broaden the microbial discussion beyond single named pathogens. Bile profiling across 10 healthy controls, 10 chronic cholecystitis patients and 5 GBC patients found no meaningful bacterial signal in normal bile. Enterobacteriaceae dominated chronic cholecystitis and GBC samples (49). Klebsiella increased across normal bile, chronic cholecystitis and GBC, and dysbiotic chronic cholecystitis was linked to larger stones, epithelial atypia and a higher inflammatory score (49). Paired mucosal metagenomics in chronic calculous cholecystitis and GBC showed differences in microbial composition, gene function, co-abundance relations and CAZyme profiles, with Peptostreptococcus stomatis and Enterococcus faecium proposed as GBC-associated species (50). These cohorts are small, but they support the idea that mucosa-associated microbes and their functions may change as chronic inflammation gives way to malignancy.

Larger bile studies have identified additional cancer-associated taxa. A 16S rRNA gene V3-V4 amplicon sequencing analysis of 30 GBC and 30 gallstone-associated chronic cholecystitis patients found that Streptococcus, Enterococcus, Halomonas, Escherichia and Caulobacter differed by disease group (12). Several Streptococcus anginosus group and Actinomyces species were higher in GBC (12). Bile multi-omics extends this observation from taxonomy to function. In carcinoma of the gallbladder, lipidome and microbial peptide signatures distinguished cancer from gallstone disease across training and validation cohorts, and bacterial taxa or functions correlated with lipid classes and bile acid profiles (11). These findings position bile as a local readout of microbial and metabolic disturbance, although diagnostic performance still requires validation across centers and sampling protocols.

The GBC-specific fungal literature currently rests on one exploratory peptide study. The initial series included bile and plasma from 10 gallstone cases, 16 GBC cases and 16 healthy liver donors, followed by an additional test series. Mass spectrometry and database assignment identified fungal peptides and a five-peptide classifier with high reported discrimination (13). The study did not culture bile fungi, confirm the organisms by ITS sequencing, or test their effects in gallbladder epithelial or tumor models. Most cancers were stage II or III, and no external validation cohort was used. The reported data define a candidate bile-based classifier in established GBC. They do not show that Aspergillus, Saccharomyces or other inferred fungi participate in tumor initiation.

## Microbial metabolites, bile acids and lipid remodeling in gallbladder disease

The gallbladder epithelium is exposed to a concentrated mixture of cholesterol, bile acids, phospholipids, bilirubin derivatives, microbial products and host inflammatory mediators. This chemical environment changes with fasting, gallbladder emptying, hepatic metabolism, intestinal reabsorption and microbial enzyme activity. In cholesterol gallstone disease, excess biliary cholesterol, altered bile acid signaling, impaired gallbladder motility, mucin secretion and local inflammation favor cholesterol crystal nucleation (5, 51). Intestinal and biliary microbes can modify this exposure by deconjugating bile acids, generating secondary bile acids, transforming dietary methylamines and altering bile lipids (14, 43). **Table 2** summarizes evidence for TMAO, individual bile acids, lipid remodeling and vesicle-contained signals.

**Table 2:** Metabolic, bile acid and lipid evidence across gallstone disease, cholecystitis and cancer.

Biological focus	Sample or model	Main observation	Interpretation and use	References
TMAO and biliary cholesterol secretion	Human cohorts, mouse strains and hepatocyte models	TMAO was associated with gallstone disease; TMAO or choline increased hepatic ABCG5/G8 and biliary cholesterol secretion.	Supports cholesterol lithogenesis. The study did not examine GBC.	(59)
TMAO, PCSK9 and APOA4	Human serum, lithogenic-diet mice and AML12 hepatocytes	TMAO increased HMGCR and ABCG5/G8, raised bile cholesterol and reduced bile acids and phospholipids; PCSK9 or APOA4 manipulation partly reversed gene changes.	Extends TMAO biology within gallstone formation. No malignant endpoint was tested.	(60)
Hypoxia, FMO3 and TMAO	Altitude-related human groups, hypoxic lithogenic-diet mice and hepatocytes	Hypoxia activated HIF-1alpha/FMO3/TMAO-related cholesterol handling and increased gallstone formation.	Many experiments used small groups and no cancer endpoint was included.	(121)
TMAO in gallstone disease and GBC	Human discovery, validation and targeted cohorts plus chow, lithogenic-diet and lithogenic-diet-plus-TMAO mice	Plasma TMAO differed across disease groups; TMAO accelerated stones, inflammation, hyperplasia and metaplasia in lithogenic-diet mice.	Age differed among human groups; targeted GBC-versus-gallstone TMAO was not significant; there was no TMAO-only group or invasive cancer.	(15)
Deoxycholic acid in GBC	Human tissue, GBC cells and xenografts	DCA was lower in GBC and suppressed tumor phenotypes through METTL3/miR-92b-3p/PTEN/PI3K-AKT signaling in the reported models.	Demonstrates that secondary bile acids should be discussed molecule by molecule.	(116)
Gut microbial and serum metabolic changes	Stool, serum and host-gene multi-omics in cholesterol gallstone disease	Patients showed microbial shifts and changes in amino acid, lipid, glucose and bile acid metabolism; UDCA and probiotic exposure were linked with lower post-bariatric gallstone risk.	Connects intestinal microbes with circulating metabolites and gallstone susceptibility.	(54)
Bile salt hydrolase-active bacteria	Fecal metagenomics and serum bile acid profiling	BSH-producing bacteria increased in gallstone patients, accompanied by higher LCA and lower TLCA.	Suggests that microbial deconjugation may reshape bile acid composition in gallstone disease.	(53)
Secondary bile acids and cholesterol-related taxa	Fecal sequencing with bile acid and cholesterol measurements	Gallstone patients had reduced microbial diversity, increased free and secondary bile acids, more 7-alpha-dehydroxylating bacteria and fewer cholesterol-lowering bacteria.	Gives earlier human support for gut microbial control of bile acid and cholesterol handling.	(52)
NAFLD and biliary bile chemistry	Bile 16S sequencing and targeted metabolomics in cholelithiasis with or without NAFLD	NAFLD changed biliary beta diversity and metabolite abundance; 12-alpha-hydroxylated bile acids correlated with Pyramidobacter, Fusobacterium and liver fibrosis markers.	Shows that systemic metabolic disease can alter bile microbiota and bile chemistry.	(57)
Stone type and bile microbiota	Bile, gallstone and plasma multi-omics	Cholesterol and pigment stone disease differed in microbial species and plasma bile acid biosynthesis signals.	Stone composition should be recorded when comparing microbial and metabolic data.	(44)
Short-chain fatty acids and prebiotics	Human fecal microbiome/metabolome and lithogenic mouse model	Cholesterol gallstone patients showed changes in butyrate, propionate, fatty acid, taurine and hypotaurine metabolism; acetate, butyrate, propionate and fructooligosaccharides reduced stones in mice.	Provides intervention-adjacent evidence for microbial metabolites in gallstone formation.	(122)
Diet-sensitive metabolites	Human diet index, serum and fecal multi-omics, genetic analyses and lithogenic mouse model	Higher dietary inflammatory index was associated with gallstone risk; lithogenic mice showed depletion of protective taxa and increases in GDCA, N-acetylarginine and long-chain acylcarnitines.	Adds diet and metabolite context to gallstone susceptibility.	(56)
Chronic cholecystitis metabolism	Serum 1H NMR metabolomics	Chronic cholecystitis showed lower glutamine, LDL/VLDL and branched-chain amino acids, with higher formate, lactate and 1,2-propanediol.	Gives a metabolic readout for the inflammatory stage between stones and cancer.	(37)
Phospholipid remodeling in tissue	Gallbladder tissue 1H NMR and computational gene analysis	Cancer tissue had higher choline-containing phospholipids than chronic cholecystitis tissue, with links to phosphatidylcholine biosynthesis and NF-kB-related genes.	Supports tissue-level lipid remodeling during malignant disease.	(62)
Bile lipidome and microbial peptides	Bile lipidomics and metaproteomics with validation cohorts	Cancer-associated bile showed fewer detected lipid species and more bacterial taxa and an eight-lipid classifier with high performance in bile and paired plasma validation.	Core bile evidence linking local lipid changes with microbial peptide signals.	(11)
Bile proteome and metabolome	Bile proteomics, metabolomics and tissue validation	Bile proteins and metabolites changed across inflammation, arachidonic acid, bile acid, amino acid, lipid and pentose phosphate pathways; 5,6-DHET had tissue correlates.	Connects bile chemistry with epithelial proliferation and injury markers.	(61)
Biliary extracellular vesicles	Biliary EV metabolomics and miRNA profiling	Gallbladder cancer EVs showed altered phosphatidylethanolamines, phosphatidylcholines, miR-181c, palmitic acid and conjugated deoxycholic acid.	Adds vesicle-contained metabolites and miRNA to the bile-based marker discussion.	(63)
Serum metabolomics in malignant disease	Untargeted serum metabolomics in gallstone disease, gallstone-associated cancer and gallstone-free cancer	Serum profiling identified disease-associated metabolites including cholic acid, glycocholic acid, glycochenodeoxycholic acid, kynurenine and glutamine.	Provides blood-based metabolic markers that can be compared with bile findings.	(64)

Bile acid metabolism provides the clearest entry point. Primary bile acids synthesized in the liver are conjugated with glycine or taurine before secretion into bile. In the intestine, bile salt hydrolase (BSH)-active bacteria deconjugate these molecules, and 7-alpha-dehydroxylating bacteria can generate secondary bile acids. Human data support this route in gallstone disease. Fecal 16S sequencing showed that gallstone patients had reduced intestinal microbial diversity, increased free and secondary bile acids, increased 7-alpha-dehydroxylating bacteria and reduced cholesterol-lowering bacteria (52). Metagenomic and bile acid profiling of 62 gallstone patients and 62 controls found enrichment of BSH-producing genera, including Bacteroides, Enterococcus, Bifidobacterium and Lactobacillaceae (53). The paired dataset also showed increased lithocholic acid and decreased taurolithocholic acid, with species-level correlations linking Bacteroides intestinalis with lithocholic acid and Bacteroides fragilis with taurolithocholic acid (53). These data point to a plausible route by which gut microbial enzymes may increase hydrophobic bile acid exposure and reduce the capacity of bile to keep cholesterol soluble.

Multi-omics studies extend bile acid analyses to amino acids, glucose metabolism, lipids and transporters. Stool and serum profiling in cholesterol gallstone patients identified changes in gut microbial composition and in amino acid, glucose, lipid and bile acid metabolism (54). Ursodeoxycholic acid, glycodeoxycholic acid and glycolithocholic acid were lower, while glycohyodeoxycholic acid and allocholic acid were higher (54). Targeted serum metabolomics identified 100 altered metabolites, including changes in amino acid, nucleotide, carbon and deoxycholic acid-related metabolism and lower ABC transporter-related pathway activity (51). Separate fecal cohorts also reported microbial and metabolic differences in gallstone disease (55, 56). Together, these measurements place amino acid availability, nucleotide turnover, oxidative stress and transporter activity alongside cholesterol saturation in gallstone biology.

Bile gives a more local readout of these changes. In bile samples from cholelithiasis patients with or without NAFLD, 16S sequencing and targeted metabolomics showed that NAFLD changed beta diversity and bile metabolite abundance (57). The comorbidity group differed in 4 phyla, 19 genera and 28 metabolites, and 12-alpha-hydroxylated bile acids correlated with Pyramidobacter, Fusobacterium, AST and FIB-4 (57). The cohort was small and bile was collected during ERCP, but the data show that systemic metabolic disease can be reflected inside the biliary lumen. Metabolic state can also change the route to stones. In bariatric surgery patients, preoperative bile acids, adipose-tissue gene expression and fecal microbial species differed between patients who did and did not develop gallstones within 2 years (58). Stone type also matters. In paired bile, gallstone and plasma profiling, cholesterol gallstone disease differed from pigment stone disease in microbial species and showed enrichment of bile acid biosynthesis in metabolomic analysis (44). Thus, bile acid findings should be interpreted with attention to stone chemistry, liver metabolic status and sampling route.

TMAO has the clearest experimental support in cholesterol gallstone formation. Gut microbes generate trimethylamine from dietary substrates, and hepatic FMO3 converts it to TMAO (59). In two human cohorts, plasma TMAO was associated with gallstone disease; mouse and hepatocyte experiments linked TMAO or choline with ABCG5/G8 expression and biliary cholesterol secretion (59). In gallstone-resistant AKR/J mice, lithogenic diet plus 0.3% TMAO produced stones in 70% of animals, whereas lithogenic diet alone produced none (59). Plasma TMAO differed across healthy, gallstone and GBC groups. In a separate experiment, female C57BL/6 mice received a lithogenic diet plus TMAO (15). Those mice developed inflammatory infiltration, hyperplasia and metaplasia, with altered ABCC3, FXR, TGR5 and IL-10 expression (15). Other mouse and hepatocyte experiments connected TMAO with PCSK9, APOA4, HMGCR, ABCG5/G8 and changes in bile cholesterol, bile acids and phospholipids (60). Across these studies, the consistent endpoint is lithogenesis. None demonstrated invasive GBC produced by TMAO.

The GBC-specific TMAO evidence comes from one human report and a lithogenic-diet mouse experiment. In the human cohorts, age increased from healthy controls to gallstone disease and GBC, targeted TMAO values did not differ significantly between GBC and gallstones (P = 0.08), and TMAO was similar in GBC with or without stones. The mouse comparison contained chow, lithogenic diet and lithogenic diet plus TMAO groups, with no TMAO-only group and no invasive cancer. Age, diet, renal function and metabolic disease can affect circulating TMAO and require adjustment. These studies consistently support TMAO-related lithogenesis. The GBC-specific observations remain limited to cross-sectional plasma measurements and preneoplastic histology in a lithogenic-diet model (15, 59, 60).

Metabolic changes are also detectable at the chronic inflammatory stage. Serum 1H NMR profiling of chronic cholecystitis showed depletion of glutamine, LDL, VLDL, alanine, branched-chain amino acids, histidine and tyrosine, with elevation of formate, lactate and 1,2-propanediol (37). The metabolic pattern points to altered energy metabolism, amino acid metabolism and inflammatory stress (37). Although serum metabolites cannot identify the exact gallbladder source, the pattern is relevant because chronic cholecystitis is the tissue setting in which stone injury, bacterial persistence and epithelial remodeling often coexist. Depleted histidine and glutamine may reflect inflammatory consumption and oxidative stress, while lactate and formate point to altered energy metabolism in inflamed tissue. These findings should be used as stage-level evidence, not as GBC-specific biomarkers.

Cancer-associated bile shows a different scale of metabolic disturbance. Bile lipidomics and metaproteomics in carcinoma of the gallbladder found reduced bile metastability, broad lipid changes, fewer detected lipid species and more bacterial taxa compared with gallstone disease and controls (11). Lipid and metaproteomic modules correlated with clinical parameters and bile acid profiles, and an eight-lipid panel classified cancer with high accuracy in bile and paired plasma validation cohorts (11). A later bile proteome and metabolome study reported 217 upregulated and 258 downregulated proteins, together with 111 upregulated and 505 downregulated metabolites in cancer-associated bile (61). The altered pathways included coagulation, arachidonic acid metabolism, bile acid metabolism, amino acid metabolism, lipid metabolism and pentose phosphate pathway, with decreases in cholesterol metabolism, PPAR, RAS, RAP1 signaling and oxidative phosphorylation (61). The metabolite panel included toluene, 5,6-DHET, creatine and phenylacetaldehyde, and bile 5,6-DHET correlated with tissue PCNA and caspase-3 (61). These studies place bile close to tumor biology, but the signals may come from tumor cells, inflamed mucosa, cholestasis, hepatocytes and microbes. Their value is highest when they are read as local molecular phenotyping, with mechanistic claims reserved for experimental follow-up.

Tissue and vesicle studies add detail to the lipid changes seen in bile. In gallbladder tissue, 1H NMR analysis found a four-fold increase in choline-containing phospholipids in GBC compared with chronic cholecystitis (62). Computational analysis linked phosphatidylcholine biosynthesis genes with RELA/NF-kB and survival in cholangiocarcinoma datasets (62). In biliary extracellular vesicles, GBC samples showed lower unsaturated phosphatidylethanolamines and phosphatidylcholines, higher miR-181c and palmitic acid, and lower conjugated deoxycholic acid, with enrichment of PI3K/AKT-related signals (63). Although the vesicle dataset is small, it suggests that bile contains packaged metabolic and miRNA signals alongside free metabolites. Serum studies point in the same direction from a less invasive sample type. Untargeted serum metabolomics distinguished gallstone-associated GBC, gallstone-free GBC and gallstone disease, with cholic acid, glycocholic acid, glycochenodeoxycholic acid, kynurenine and glutamine among prominent altered metabolites (64).

Metabolic studies in this field answer two separate questions. Perturbation experiments test whether a molecule changes cholesterol solubility, bile transport, epithelial barrier function or inflammatory signaling. Biomarker studies test whether bile or blood can distinguish GBC from inflammatory gallbladder disease. A classifier may reflect tumor growth, cholestasis or systemic inflammation without participating in tumor initiation. Conversely, a molecule with limited diagnostic performance may still alter gallstone biology in a controlled model. Study design and endpoint should accompany every metabolic claim.

## Inflammatory and immune remodeling from chronic cholecystitis to gallbladder cancer

Gallbladder tissue studies are most informative when the sampled lesion and study design are explicit. RNA-seq of three normal gallbladders, four with gallstone-related chronic inflammation, five early GBCs and five advanced GBCs identified different pathway signals across the four groups (36). NSAID use and immune-related proteins were associated with dysplasia among symptomatic gallstone patients (65). Salmonella infection, H. pylori CagA and TMAO exposure produced epithelial or inflammatory changes in experimental systems (15, 16, 35). The experiments document specific injury responses in the tested models. They do not constitute observation of the same patient moving from cholecystitis to GBC.

Single-cell studies compare tissue states at surgery. One scRNA-seq study analyzed 24 samples: six chronic cholecystitis specimens, 12 treatment-naive primary GBCs and six matched metastases. It identified resting, immune-regulating and gastrointestinal metaplastic cholangiocytes among non-malignant epithelial cells (66). Protein profiling and cell-resolved datasets describe inflammatory signaling, progenitor-like malignant cells, tumor-associated neutrophils, macrophages and metabolically distinct epithelial states (67–69). These cross-sectional comparisons define cellular differences among sampled tissues; they do not follow the same patients from inflammation through malignancy.

Malignant epithelium can actively shape immune suppression. ErbB pathway-mutated tumors contain larger epithelial subtype 1/2 populations, more regulatory T cells and more M2 macrophages (70). Tumor-derived MDK interacts with LRP1 on macrophages, while macrophage CXCL10 is linked with CXCR3-expressing regulatory T cells (70). OLFM4 has been identified as an immune regulator and later linked with immunosuppressive features and progression (71, 72). ERRalpha-ETV5 signaling drives PD-L1 upregulation, and B7-family ligand expression tracks with tumor heterogeneity and prognosis (73, 74).

Myeloid cells repeatedly appear as major immune partners of gallbladder tumor cells. Infiltrating macrophage-derived YKL-40 cooperated with GDF15 to maintain an immunosuppressive tumor environment (75). M2-like macrophage-derived CCL2, macrophage-rich liver-invasive regions and liver-metastasis scRNA-seq further linked macrophages with stemness, metastasis and T-cell dysfunction (76–78). Perturbation and spatial localization provide stronger support than marker lists alone.

T-cell data separate lymphocyte abundance from effective antitumor activity. Single-cell analysis of infiltrating T cells identified exhausted cytotoxic populations and candidate therapeutic targets (79). Co-expression of PD-1 and TIM-3 was associated with poor prognosis, and clinical cohorts have evaluated predictors of immune-checkpoint inhibitor response in GBC (80, 81). PD-1 blockade scRNA-seq and spatial analyses further describe treatment-associated immune states, precursor-to-cancer regions and distinct immunophenotypes (82–84).

Spatial transcriptomics remains sparsely sampled. Two gallbladders containing normal epithelium, low-grade BilIN, high-grade BilIN and adenocarcinoma were profiled across 24 and 32 regions of interest (83). Cell-resolved analysis of gallbladder neck or cystic duct carcinoma identified a LAMB3-associated immunosuppressive environment (85). Fibroblast and endothelial studies linked extracellular-matrix remodeling and CD34-positive CD90-positive endothelial cells with adenoma-to-carcinoma differences and metastasis (86, 87). Population-specific immunogenomic analysis identified immune features with prognostic relevance in GBC (88). The two-patient spatial analysis permits within-specimen comparison, but it does not define a general disease chronology.

Metabolism also influences immune and stromal behavior. Amino acid metabolism has been proposed to affect malignant phenotypes and macrophage polarization in biliary tumors (89). Cell-resolved and functional studies identify epithelial-macrophage interactions and OLFM4-dependent immune suppression in GBC (69, 72). These processes may influence invasion and treatment response, but their relevance to chronic cholecystitis has not been established.

Single-cell and spatial data are strongest for established GBC, where they identify epithelial, myeloid, lymphoid, stromal and endothelial states. Early lesions remain thinly sampled. The largest single-cell study included 1,117,245 cells from 102 patients, but the 16 non-tumor gallbladders included only two low-grade and one high-grade neoplasia; the remaining samples were xanthogranulomatous or chronic cholecystitis (68). Macrophage-rich invasive regions and LAMB3-associated immune suppression have been described in established GBC (77, 85). Paired collection of bile, stones, adjacent mucosa and tumor tissue from the same patient would allow these cell-level findings to be compared with local microbial and metabolic measurements.

## Biomarker translation and early detection in gallbladder disease

Early detection is where GBC research most directly meets clinical need. Most patients present with vague symptoms, advanced local invasion or incidental cancer after cholecystectomy. Imaging and serum markers have limited sensitivity for distinguishing inflammatory gallbladder disease from malignancy, especially before surgery (90, 91). Marker development has the clearest role in patients already undergoing evaluation for a gallbladder lesion, not in population screening (92, 93).

Bile directly samples the luminal environment of the gallbladder. Bile lipidomics and metaproteomics distinguish carcinoma from gallstone disease in selected training and validation cohorts (11). Bile proteins and metabolites, including toluene, 5,6-DHET, creatine and phenylacetaldehyde, also differed between GBC and inflammatory controls (61). The fungal peptide panel reported high discrimination in a small internal analysis, but the organisms were not confirmed by culture or ITS sequencing and the panel has not undergone external validation (13). These studies support local classification signals and do not establish that the measured molecules or inferred fungi initiate GBC.

Blood-based metabolite and protein assays are easier to obtain before surgery and can be repeated over time. Untargeted serum metabolomics in gallstone-free GBC, gallstone-associated GBC and gallstone disease identified bile acid, kynurenine and glutamine-related signatures (64). Plasma proteomic profiling of 82 patients with suspected GBC identified proteins associated with postoperative GBC versus cholecystitis, and an internally tested subset achieved 94% accuracy (94). Routine serum markers, including CA19-9, CEA and CA242, have also been evaluated in GBC, but reported performance varies with disease stage and comparator selection (90–92).

Comparisons with cholangiocarcinoma (CCA) and pancreatic ductal adenocarcinoma (PDAC) help test whether a candidate signal reflects gallbladder cancer, biliary obstruction or malignant disease more generally. In a treatment-naive, fasting ERCP-bile study of 336 patients, a six-metabolite panel separated gallbladder cancer from benign gallbladder disease with AUCs of 0.951 in the exploratory cohort and 0.924 in the validation cohort; the corresponding CCA-versus-benign biliary comparison yielded AUCs of 0.833 and 0.804 (95). These results extend bile-based classification, but they do not make individual metabolites cancer-specific. In a direct comparison of extrahepatic CCA, choledocholithiasis and post-surgical bile-duct injury controls, phosphatidylcholine depletion occurred in both extrahepatic CCA and stones. Acylcarnitines were more prominent in stone disease, whereas selected diacylglycerols and lower lysophosphatidylcholine (LPC) species characterized extrahepatic CCA (96). The study included 33 participants and did not provide an adjusted diagnostic model. A six-lipid panel tested in three CCA cohorts retained high AUCs in the first two cohorts, 0.943 and 0.956, but fell to 0.828 in the third, with 62.5% sensitivity and 91.8% specificity (97). Targeted plasma profiling of 42 CCA cases and 120 patients with benign biliary disease, 106 with gallstone-related disorders, produced a model based on chenodeoxycholic acid (CDCA) and taurochenodeoxycholic acid with an AUC of 0.970 in a randomly split same-center validation set (98). However, CCA had substantially higher bilirubin and alkaline phosphatase (ALP), the measured bile acids correlated with these variables, and the model did not adjust for them. The result therefore indicates discrimination in that cohort, without establishing a tumor-specific signal or external clinical validation.

Direct comparisons between distal CCA and PDAC showed broad overlap in circulating metabolism. Both cancers had higher serum triglycerides, oxidized fatty acids and bile acids and lower sphingomyelins and glycerophosphatidylcholines; a nine-metabolite panel provided moderate site discrimination, with an AUC of 0.82 in validation (99). Adding CA19–9 to the metabolite panel produced a pooled AUC of 0.888, with 71.4% sensitivity and 89.2% specificity. Bile profiling likewise identified lower phosphatidylcholines, ether-linked phosphatidylcholines, sphingomyelins, ceramides and glycine-conjugated bile acids in both cancers, whereas higher bile glucose was more prominent in PDAC (100). The reported bile classifiers were assessed after synthetic observations had been generated from the same patient cohort, so the measured differences are more informative than the reported accuracy. These findings make shared lipid and bile-acid changes poor stand-alone indicators of tumor site; bile glucose and selected lipid combinations remain candidate differentiators.

Studies that measure microbiota and metabolites in the same bile sample provide a direct way to examine their co-occurrence. Profiling of 13 CCA, 34 benign biliary disease and 12 primary sclerosing cholangitis samples identified methylguanine, butyric acid and CDCA as a candidate combination; bacterial associations also varied with papilla status, antibiotic exposure, ursodeoxycholic acid use and serum bilirubin (101). Intraoperative lower-bile-duct sampling from 15 PDAC and 12 benign cases linked Muribaculum abundance with PA(10:0/a-17:0), but the combined classifier was assessed by seven-fold internal cross-validation and the PDAC group had greater obstruction (102). These observations support matched microbial and metabolite measurements, extraction blanks and complete records of fasting, sampling route, obstruction, drainage, stenting, antibiotics and treatment. They do not establish that a named organism produced a measured metabolite. Prospective cohorts should compare gallstone disease, chronic cholecystitis, gallbladder cancer, CCA and PDAC under matched sampling conditions and test added value beyond imaging and CA19-9. **Figure 1** places these candidate metabolomic signals in relation to gallstones, microbial biofilms and epithelial injury.

Liquid biopsy studies extend this work to nucleic acids and extracellular-vesicle markers. Targeted cfDNA sequencing in paired serum and tissue from 40 GBC cases, with 20 cholecystitis and four non-inflamed gallbladder controls, identified recurrent variants; paired cfDNA and tissue shared 66.2% of detected genetic abnormalities (103). A five-miRNA plasma exosome panel was validated in 102 GBC and 112 chronic cholecystitis cases from several centers, with an AUC of 0.905 overall and 0.802 for early-stage GBC (104). In a separate serum study, miR-183-5p and miR-3651 showed more modest discrimination (105).

Circulating RNA studies have produced different marker sets across platforms and cohorts. Plasma miR-146a showed diagnostic value in a pilot cohort that included 20 GBC, 20 cholelithiasis and 20 healthy-control participants (106). Serum miR-1, miR-130, miR-146, miR-182 and miR-21 were measured in a separate study of GBC, cholecystitis and control samples (107). Serum lncRNA panels add another candidate class, but differences in sample handling, disease controls and stage distribution limit comparison across circulating RNA studies (108, 109).

These data are most relevant to preoperative assessment of patients already being evaluated for gallbladder disease. Bile markers can be studied when biliary access is clinically indicated, while blood tests are more practical for broader triage. Prospective studies should test whether multimarker panels improve discrimination beyond imaging, CA19–9 and routine pathology (90, 92). Bile lipidomics provides one example in which a signal discovered in bile was subsequently tested in paired plasma (11) (**Figure 2**; **Table 3**).

**Figure 2:**
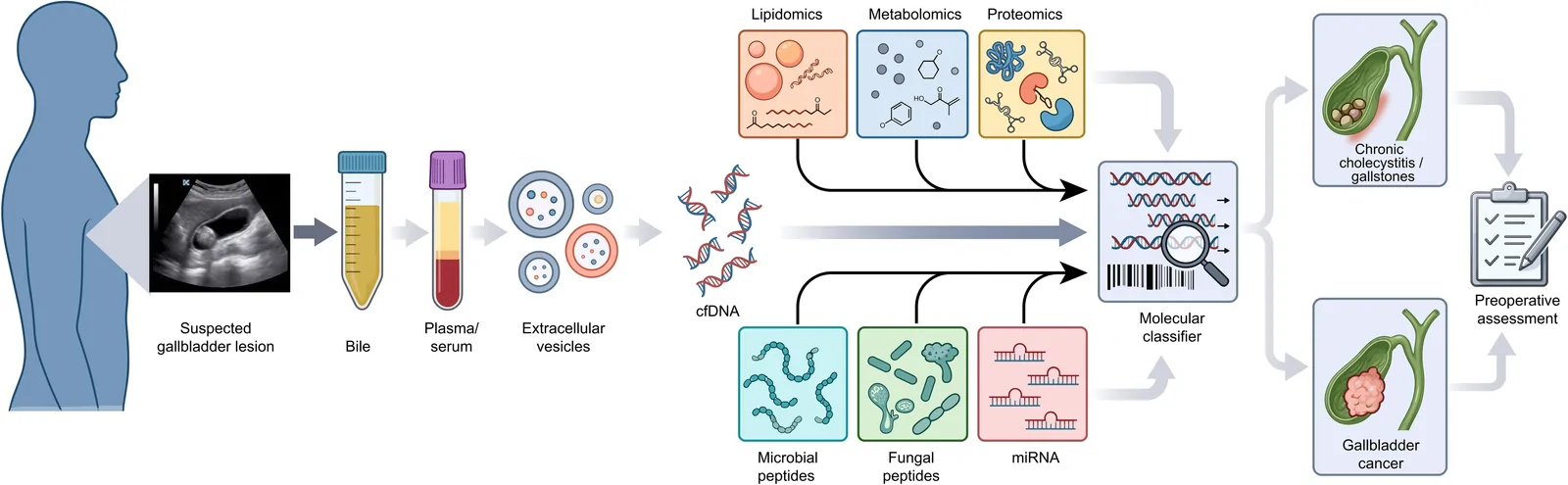
Bile- and blood-based multi-omics for preoperative assessment in suspected gallbladder cancer. Patients with suspicious gallbladder lesions can be evaluated with local and circulating samples, including bile, plasma or serum, extracellular vesicles and cfDNA. Lipidomics, metabolomics, proteomics, microbial peptide profiling, fungal peptide profiling, miRNA assays and cfDNA sequencing may support molecular classification. The practical aim is to distinguish chronic cholecystitis or gallstone disease from GBC before definitive surgery.

**Table 3:** Candidate molecular markers for preoperative evaluation.

Marker class	Sample	Study design and sample size	Reported performance (95% CI where available)	Validation and limitations	References
Routine serum markers	Serum	Narrative review; no diagnostic cohort	CEA and CA19–9 remain widely used, but sensitivity for early GBC is limited; no single pooled estimate was reported.	Clinical comparators, not validated stand-alone early-detection tests.	(90)
CA242	Serum	Prospective cross-sectional study; n=80 (50 GBC, 20 chronic calculous cholecystitis, 10 healthy controls)	AUC 0.756 (95% CI, 0.651-0.862), sensitivity 64.0%, specificity 93.3%.	Single-center exploratory study; 76% of GBC cases were late stage; no external validation.	(91)
Multi-parameter clinical markers	Serum and clinical data	Retrospective study; n=90, including 6 adenocarcinomas and 7 metaplasias	CEA AUC 1.000 (reported 95% CI, 1.000-1.000); CA19–9 AUC 0.968 (reported 95% CI, 0.932-1.005).	The small event counts make the performance estimates unstable; no independent validation.	(92)
Plasma proteomics	Plasma	Retrospective single-center study; n=82 (44 GBC, 38 cholecystitis); 80:20 internal split	An eight-protein subset achieved 94% test-set accuracy; 95% CI not reported.	Clinically relevant comparator, but only internal testing in a small cohort; no external validation.	(94)
Plasma exosomal miRNA	Plasma exosomes	Three-stage study; multicenter validation n=214 (102 GBC, 112 chronic cholecystitis)	Five-miRNA panel: validation AUC 0.905, sensitivity 81.37%, specificity 86.61%; early-stage AUC 0.802. Training AUC 0.920 (95% CI, 0.820-0.974).	The prespecified training cutoff was tested across several centers; an independent implementation cohort is still needed.	(104)
cfDNA mutation panel	Serum cfDNA and matched tissue	Prospective case-control series; n=64 (40 GBC, 20 cholecystitis, 4 non-inflamed controls)	Paired cfDNA and tissue shared 66.2% of alterations; TP53 gene-level sensitivity 94.44% and specificity 100%; 95% CIs not reported.	Gene-level estimates and four non-inflamed controls limit precision; no external validation.	(103)
Plasma miR-146a and miR-19a	Plasma and tissue	Pilot observational study; n=60 (20 GBC, 20 cholelithiasis, 20 healthy controls)	Plasma miR-146a AUC 0.953, sensitivity 80%, specificity 95%; 95% CI not reported.	Single-center pilot; 65% of GBC cases were stage IV; no external validation.	(106)
Serum miR-183-5p and miR-3651	Serum	Single-center observational study; serum n=130 (50 GBC, 50 gallstone, 30 healthy controls)	AUC 0.684 for miR-183-5p, 0.752 for miR-3651 and 0.720 combined; 95% CIs not reported.	Modest discrimination and no external validation; tissue discovery used 3 GBC and 3 normal samples.	(105)
Serum lncRNAs	Matched serum and tissue	Case-control study; n=128 (63 GBC, 19 cholecystitis, 46 normal controls)	Against normal controls, AUCs were 0.75-0.96 across five lncRNAs; combined sensitivity 84.13%-100% with reported 95% CIs.	The highest AUC was obtained against normal controls; disease-control performance and external validation remain limited.	(109)
Genotype-linked lncRNA expression	Tissue, serum eQTL and genotype data	Three datasets: tissue preselection n=93; serum eQTL validation n=93; risk association 540 GBC and 2,397 controls	Predicted LINC00662 expression associated with GBC risk: OR 1.25 per log2 unit (95% CI, 1.04-1.52).	A susceptibility study based on genotype-predicted expression, not a direct diagnostic serum assay.	(123)
Bile fungal peptides	Bile and plasma	Bile discovery n=42 (10 gallstone, 16 GBC, 16 healthy donors); machine-learning analysis across n=82	Five-peptide panel AUC 0.969 in internal analysis; 70:30 split; 95% CI not reported.	No culture or ITS confirmation, no epithelial experiment and no external validation.	(13)
Bile-to-plasma metabolite panel	Bile and paired plasma	Training bile cohort n=87; separate validation n=80 with bile and paired plasma	Four-metabolite panel: 99% accuracy and >98% sensitivity/specificity; 95% CIs not reported.	Separate validation cohorts were used, but no independent external-center validation was reported.	(61)
Bile lipid panel	Bile and paired plasma	Training bile cohort n=87; validation n=38 with bile and paired plasma	Eight-lipid panel: 99% accuracy and >98% sensitivity/specificity; 95% CIs not reported.	The validation cohorts were small and came from the same study setting; external-center validation is absent.	(11)
miRNA biosensors	Electrochemical platforms	Technology review; no clinical diagnostic cohort	Most platforms used synthetic or spiked matrices; no patient-level accuracy estimate was available.	Assay-engineering evidence; clinical validation is absent.	(124)

AUC, area under the receiver operating characteristic curve; cfDNA, cell-free DNA; CI, confidence interval; GBC, gallbladder cancer; lncRNA, long non-coding RNA; NGS, next-generation sequencing. Internal validation denotes resampling or a split within the same study population; external validation denotes testing in an independent population or center.

## Future directions and conclusions

The reviewed literature spans bile microbiota, tumor tissue and integrated microbiome-metabolomics studies. These materials answer different questions. Bile records soluble lipids, bile acids, microbial products and vesicles; stones retain adherent microorganisms and biofilms; mucosa shows epithelial injury and inflammation; tumor tissue defines established malignant and immune states. Paired collection from the same patient can compare local exposure with tissue response without combining unrelated cohorts.

Low-biomass bile studies require negative controls and detailed procedural metadata. Antibiotics, biliary drainage, ERCP, surgical timing, bile leakage and DNA extraction can alter microbial yield (8, 10). Future cohorts should report these variables and the exact anatomic source of each specimen. Bile, stones, mucosa and tumor tissue should not be treated as interchangeable samples.

Disease stage requires equal precision. Comparisons between gallstone disease and advanced GBC are useful for marker discovery, but they provide limited information about the interval in which chronic inflammation and dysplasia emerge (3, 90). Cholecystectomy cohorts can examine postoperative gut and metabolic changes, while xanthogranulomatous cholecystitis provides a clinically relevant inflammatory mimic of GBC (110–112). Microbial bile acid studies should report the organisms, enzymes and individual bile acid species involved (113).

Clinical phenotyping should accompany molecular profiling. Large cholecystectomy series from high-incidence regions can define the prevalence and distribution of dysplasia and incidental GBC (114). Stone number, size and composition, wall thickness, mucosal dysplasia, xanthogranulomatous inflammation, diabetes, NAFLD, prior biliary procedures and antibiotic exposure all affect interpretation. These variables determine whether a sample represents common gallstone disease, persistent inflammation or a lesion close to malignant transformation. Prospective recording will make small cohorts more informative and clarify why reported microbial taxa differ among studies.

Metabolite studies should name the individual molecule and measured endpoint. Experimental manipulation of TMAO and stigmasterol alters cholesterol gallstone biology through distinct metabolic pathways (15, 115). For TMAO, the GBC evidence is cross-sectional and the mouse histology stops at metaplasia (15). Deoxycholic acid was lower in GBC and suppressed tumor phenotypes in one cell and xenograft study (116), so secondary bile acids should not be treated as a uniform tumor-promoting class. Cancer-associated bile lipid and peptide signatures remain candidates for diagnosis and require biological testing of selected molecules (11, 13, 61).

Perturbation models should match the organism and measured endpoint. S. Typhimurium in lithogenic mice and enterohepatic Helicobacter species in murine gallstone models address different infections and outcomes (16, 117). Neither model estimates GBC risk associated with chronic human S. Typhi carriage. TMAO exposure and exosome-derived miR-107 have been tested for effects on cholesterol secretion and gallstone progression (15, 118). These experiments concern gallstone biology and should be separated from malignant endpoints. Gallbladder cholangiocyte organoids can test controlled epithelial responses, while matched primary tumor and patient-derived xenograft models can assess established cancer biology (119, 120).

Single-cell and spatial analyses should be interpreted according to sampling depth. Large and specialized datasets define primary GBC, invasive-margin biology and gallbladder neck or cystic duct carcinoma (68, 77, 85). Precursor sampling remains limited to two HGIN samples in one scRNA-seq study (69), two spatially profiled gallbladders (83), and three neoplastic lesions in the 102-patient study (68). Pseudotime orders transcriptional states and should be reported as a computational result. Longitudinal inference will require prospective clinical sampling or archival series with well-defined histopathology. Paired bile, stone, mucosal and tumor measurements within the same patient can then test whether a local exposure co-occurs with a defined epithelial or immune state.

Clinical translation should begin with patients already being assessed for a gallbladder lesion. Bile lipidomics and microbial peptides, plasma proteomics and exosomal miRNAs can be evaluated with imaging, clinical variables and CA19-9 (11, 94, 104). Fungal peptides remain a pilot component (13). Prospective studies should compare these panels across chronic cholecystitis, xanthogranulomatous inflammation, early GBC, CCA and PDAC. They should record biliary obstruction and sampling conditions and test whether each panel adds value beyond imaging and CA19-9.

Gallstone-associated GBC can be studied with a staged, sample-matched design: bile and stones for exposure, mucosa for epithelial injury, and tumor tissue for established malignant organization. Salmonella persistence and TMAO-related lithogenesis have experimental support, while fungal findings and several multi-omics markers remain exploratory. Relevant gallstone and inflammatory controls, paired local samples and endpoints matched to each claim will distinguish common stone disease from persistent mucosal injury and established GBC. **Table 4** summarizes the principal finding, strongest evidence, main limitation and interpretive consequence for each major section.

**Table 4:** Major findings, evidence strengths and remaining limitations across the review.

Review section	Major finding	Strongest evidence	Main limitation	Interpretive consequence	Representative references
Gallstones, chronic inflammation and GBC	GBC often occurs with stones and chronic inflammation, but progression among patients with stones is uncommon.	Epidemiology, cholecystectomy pathology and human tissue series	Few prospective samples of metaplasia or dysplasia; pathology series do not follow individual lesions.	Gallstones mark a common background; added local and tissue features are needed to identify lesions nearer malignancy.	(24, 32, 46)
Biliary microbiota and microbial persistence	Stones can retain bacteria and biofilms; Salmonella has the most developed disease-relevant evidence.	Human carriage studies, stone or bile detection and experimental biofilm models	Gallstone confounding, low biomass, procedural contamination and sparse fungal evidence.	Report sample type, collection route, antibiotic exposure and drainage status with microbial findings.	(31, 116, 121, 125)
Metabolic changes in gallstone disease	Cholesterol saturation, bile-acid transformation, phospholipid remodeling and TMAO-related cholesterol handling contribute to stone biology.	Human metabolomics plus controlled mouse or cell experiments	Several experiments end at stones or metaplasia and do not test invasive GBC.	A metabolic effect on gallstones should be separated from evidence about malignant disease.	(44, 46, 54)
Epithelial, immune and stromal changes	Established GBC contains stressed epithelium, recruited myeloid cells, exhausted T cells and remodeled stroma.	Bulk RNA sequencing, scRNA-seq and spatial analyses of human tissue	Preneoplastic lesions are rare; computational ordering is not clinical follow-up.	Cell-resolved findings are strongest for established tumors and need testing in defined precursor lesions.	(20, 47)
Preoperative biomarkers	Bile lipid, metabolite and peptide signals, plus circulating proteins or nucleic acids, can separate GBC from inflammatory gallbladder disease in selected cohorts.	Studies with gallstone or cholecystitis controls and separate validation sets	Many panels are single-center, internally tested or sensitive to stage and obstruction.	Compare early GBC with chronic and xanthogranulomatous cholecystitis and assess added value beyond imaging and CA19-9.	(11, 95, 106)
Specificity across pancreaticobiliary disease	Phosphatidylcholine or LPC depletion and altered bile acids recur in stones, CCA and PDAC; selected acylcarnitines, diacylglycerols, glucose and lipid combinations may aid differential diagnosis.	Direct multi-disease bile or serum comparisons	Cholestasis, drainage, treatment, cohort composition and limited external validation can dominate classifier performance.	Separate shared metabolites from candidate discriminators and test them under matched sampling conditions.	(96–100)

## Glossary

5,6-DHET: 5,6-dihydroxyeicosatrienoic acid
ABCC3: ATP-binding cassette subfamily C member 3
ALP: alkaline phosphatase
AUC: area under the receiver operating characteristic curve
BSH: bile salt hydrolase
CA125: cancer antigen 125
CA19-9: carbohydrate antigen 19-9
CA242: carbohydrate antigen 242
CAGB: carcinoma of the gallbladder
CCA: cholangiocarcinoma
CDCA: chenodeoxycholic acid
CEA: carcinoembryonic antigen
cfDNA: cell-free DNA
CODEX: co-detection by indexing
CRP: C-reactive protein
ERCP: endoscopic retrograde cholangiopancreatography
EVs: extracellular vesicles
FIB-4: fibrosis-4 index
FMO3: flavin-containing monooxygenase 3
FTIR: Fourier-transform infrared spectroscopy
FXR: farnesoid X receptor
GBC: gallbladder cancer
GCDCA: glycochenodeoxycholic acid
GDCA: glycodeoxycholic acid
ITS2: internal transcribed spacer 2
KDM6B: lysine demethylase 6B
LCA: lithocholic acid
LDL: low-density lipoprotein
lncRNA: long non-coding RNA
LPC: lysophosphatidylcholine
LXR: liver X receptor
miRNA: microRNA
NF-kB: nuclear factor-kappa B
NGS: next-generation sequencing
OCLN: occludin
PCNA: proliferating cell nuclear antigen
PDAC: pancreatic ductal adenocarcinoma
PDX: patient-derived xenograft
RNA-seq: RNA sequencing
ROS: reactive oxygen species
scRNA-seq: single-cell RNA sequencing
TGR5: Takeda G protein receptor 5
TLCA: taurolithocholic acid
TMAO: trimethylamine N-oxide
Tregs: regulatory T cells
UDCA: ursodeoxycholic acid
VLDL: very-low-density lipoprotein
WES: whole-exome sequencing
XGC: xanthogranulomatous cholecystitis
ZO-1: zonula occludens-1
