# Supplementary material for: Biliary microbiota and metabolic reprogramming in gallstone-associated gallbladder cancer: evidence from bile, stones, mucosa and tumor tissue
Source: Front Oncol. 2026 Sep 25;16:1925531. doi: 10.3389/fonc.2026.1925531 (PMC13650614; doi:10.3389/fonc.2026.1925531)
Supplement: Supplementary file 1 [file Table1.docx]

**Supplementary Table S1. Literature search strategies**

| **Database** | **Topic** | **Date range** | **Exact search string** | **Records retrieved** |
| --- | --- | --- | --- | --- |
| PubMed | salmonella gallbladder cancer | 1900-01-01 to 2026-08 | ("Gallbladder Neoplasms"[Mesh] OR "gallbladder cancer"[tiab] OR "gallbladder carcinoma"[tiab] OR "gallbladder neoplasm"[tiab] OR "biliary tract cancer"[tiab]) AND ("Salmonella"[Mesh] OR Salmonella[tiab] OR typhoid[tiab] OR "S. Typhi"[tiab] OR "S. Typhimurium"[tiab]) | 78 |
| PubMed | salmonella carriage gallstones biofilm | 1900-01-01 to 2026-08 | (Salmonella[tiab] OR typhoid[tiab] OR "chronic carrier"[tiab]) AND (gallstone*[tiab] OR cholelithiasis[tiab] OR cholecystitis[tiab] OR gallbladder[tiab] OR bile[tiab]) AND (carriage[tiab] OR persistence[tiab] OR biofilm[tiab] OR cancer[tiab] OR carcinoma[tiab]) | 264 |
| PubMed | biliary fungi mycobiome | 1900-01-01 to 2026-08 | ("Gallbladder Diseases"[Mesh] OR "Gallbladder Neoplasms"[Mesh] OR gallbladder[tiab] OR biliary[tiab] OR bile[tiab] OR gallstone*[tiab]) AND (mycobiome[tiab] OR mycobiota[tiab] OR fungi[tiab] OR fungal[tiab] OR Candida[tiab] OR "fungal peptide"[tiab]) | 1016 |
| PubMed | tmao biliary disease | 1900-01-01 to 2026-08 | ("trimethylamine N-oxide"[tiab] OR "trimethylamine-N-oxide"[tiab] OR TMAO[tiab]) AND (gallstone*[tiab] OR cholelithiasis[tiab] OR cholecystitis[tiab] OR gallbladder[tiab] OR biliary[tiab] OR bile[tiab]) | 414 |
| PubMed | tmao gallbladder malignancy | 1900-01-01 to 2026-08 | ("trimethylamine N-oxide"[tiab] OR "trimethylamine-N-oxide"[tiab] OR TMAO[tiab]) AND ("gallbladder cancer"[tiab] OR "gallbladder carcinoma"[tiab] OR "gallbladder tumor"[tiab] OR "gallbladder tumour"[tiab] OR "Gallbladder Neoplasms"[Mesh]) | 2 |
| PubMed | single cell spatial gallbladder | 1900-01-01 to 2026-08 | ("single-cell"[tiab] OR "single cell"[tiab] OR scRNA-seq[tiab] OR scRNAseq[tiab] OR "spatial transcriptomics"[tiab] OR "spatial proteomics"[tiab]) AND ("gallbladder cancer"[tiab] OR "gallbladder carcinoma"[tiab] OR "gallbladder neoplasm"[tiab] OR "chronic cholecystitis"[tiab] OR "gallbladder dysplasia"[tiab] OR "gallbladder intraepithelial neoplasia"[tiab]) | 46 |
| PubMed | biliary microbiome metabolomics gbc | 1900-01-01 to 2026-08 | ("gallbladder cancer"[tiab] OR "gallbladder carcinoma"[tiab] OR "Gallbladder Neoplasms"[Mesh]) AND ("biliary microbiome"[tiab] OR "bile microbiome"[tiab] OR "biliary microbiota"[tiab] OR microbiota[tiab] OR microbiome[tiab] OR metabolomics[tiab] OR lipidomics[tiab] OR multiomics[tiab] OR "multi-omics"[tiab]) | 67 |
| PubMed | bile acids lipids gbc | 1900-01-01 to 2026-08 | ("gallbladder cancer"[tiab] OR "gallbladder carcinoma"[tiab] OR "Gallbladder Neoplasms"[Mesh]) AND ("bile acid"[tiab] OR "bile acids"[tiab] OR "secondary bile acid"[tiab] OR deoxycholate[tiab] OR "deoxycholic acid"[tiab] OR cholesterol[tiab] OR lipidomics[tiab] OR "lipid metabolism"[tiab] OR "metabolic reprogramming"[tiab]) | 331 |
| PubMed | cholecystitis dysplasia gbc | 1900-01-01 to 2026-08 | ("chronic cholecystitis"[tiab] OR gallstone*[tiab] OR cholelithiasis[tiab]) AND (metaplasia[tiab] OR dysplasia[tiab] OR "intraepithelial neoplasia"[tiab] OR carcinogenesis[tiab]) AND (gallbladder[tiab] OR "gallbladder cancer"[tiab] OR "gallbladder carcinoma"[tiab]) | 347 |
| PubMed | recent integrative reviews | 1900-01-01 to 2026-08 | ("gallbladder cancer"[tiab] OR "gallbladder carcinoma"[tiab] OR "Gallbladder Neoplasms"[Mesh]) AND (microbiome[tiab] OR microbiota[tiab] OR metabolomics[tiab] OR "bile acid"[tiab] OR "metabolic reprogramming"[tiab]) AND (Review[ptyp] OR review[ti]) | 31 |
| PubMed | biliary low biomass methods | 1900-01-01 to 2026-08 | (bile[tiab] OR biliary[tiab] OR gallbladder[tiab]) AND (microbiome[tiab] OR microbiota[tiab]) AND ("low biomass"[tiab] OR contamination[tiab] OR "negative control"[tiab] OR sampling[tiab] OR reproducibility[tiab]) | 124 |
| Crossref | salmonella gallbladder | inception to 2026-08 | Salmonella typhoid gallbladder cancer gallstones | 100 |
| Crossref | biliary mycobiome | inception to 2026-08 | bile gallbladder mycobiome fungal gallstones cancer | 100 |
| Crossref | tmao gallbladder | inception to 2026-08 | TMAO trimethylamine N-oxide gallstones gallbladder cancer | 100 |
| Crossref | single cell gallbladder | inception to 2026-08 | single-cell spatial gallbladder cancer cholecystitis dysplasia | 100 |
| Crossref | integrative reviews | inception to 2026-08 | gallbladder cancer biliary microbiome metabolomics review | 100 |
